# Medical Lexicon of Modern Terminology; Being a Complete Vocabulary of Definitions, Including All the Technical Terms Employed by Writers and Teachers of Medical Science at the Present Day, and Comprising Several Hundreds of Words Not Found in Any Other Dictionary

**Published:** 1855-09

**Authors:** 


For sale by PHINNEY & Co.

---

ART. VIII.—*Medical Lexicon of Modern Terminology; being a complete Vocabulary of Definitions, including all the technical terms employed by writers and teachers of Medical Science at the present day, and comprising several hundreds of words not found in any other dictionary. Third Edition.* By D. MEREDITH REESE, M. D., LL. D., Resident Physician of Bellevue Hospital, N. Y., Editor of Cooper's Surgical Dictionary, &c., &c. New York: S. S. & Wm. Wood, 261 Pearl St. 1855.

The fact of a third edition implies that this little handbook of medical terms has found favor with the profession. Its own preface expresses so well its design and object, that we can best inform our readers of its claims by quoting from it:

"The design being to bring this Lexicon within the smallest possible compass, and adapt it to the use of students, as a Pocket Companion, the briefest possible definition has been given in every case, consistent with perspicuity; omitting all reference to the etymology of the terms, for which larger works may be consulted, and which will still be needed in every medical library. In the department of derivation, the late work of Professor Dunglison leaves nothing to be desired.

"It is simply as a vocabulary of definitions that the present vade-mecum is commended to the profession and the public, without any claim of novelty or other merit, except convenience, brevity, simplicity, and accuracy. If in these attributes it shall be deemed worthy of approval, it cannot fail to be useful as a help to students and junior practitioners, for whose benefit it has been prepared, and to whom it is affectionately inscribed by the author."

